# Pushing and Pulling on Ropes: Hierarchical Woven Materials

**DOI:** 10.1002/advs.202001271

**Published:** 2020-08-24

**Authors:** Widianto P. Moestopo, Arturo J. Mateos, Ritchie M. Fuller, Julia R. Greer, Carlos M. Portela

**Affiliations:** ^1^ Division of Engineering and Applied Science California Institute of Technology Pasadena CA 91125 USA; ^2^ Independent Artist Newport News VA 23601 USA; ^3^ Department of Mechanical Engineering Massachusetts Institute of Technology Cambridge MA 02139 USA

**Keywords:** architected materials, extensible materials, resilient materials, tensile responses, woven lattices

## Abstract

Hierarchy in natural and synthetic materials has been shown to grant these architected materials properties unattainable independently by their constituent materials. While exceptional mechanical properties such as extreme resilience and high deformability have been realized in many human‐made three‐dimensional (3D) architected materials using beam‐and‐junction‐based architectures, stress concentrations and constraints induced by the junctions limit their mechanical performance. A new hierarchical architecture in which fibers are interwoven to construct effective beams is presented. In situ tension and compression experiments of additively manufactured woven and monolithic lattices with 30 µm unit cells demonstrate the superior ability of woven architectures to achieve high tensile and compressive strains (>50%)—without failure events—via smooth reconfiguration of woven microfibers in the effective beams and junctions. Cyclic compression experiments reveal that woven lattices accrue less damage compared to lattices with monolithic beams. Numerical studies of woven beams with varying geometric parameters present new design spaces to develop architected materials with tailored compliance that is unachievable by similarly configured monolithic‐beam architectures. Woven hierarchical design offers a pathway to make traditionally stiff and brittle materials more deformable and introduces a new building block for 3D architected materials with complex nonlinear mechanics.

## Graphical abstract

An architected material design in which fibers are interwoven to construct effective beams is presented. Mechanical characterization of additively manufactured samples and numerical analysis reveal the superior ability of woven architectures to achieve high tensile and compressive strains while accruing minimal damage, opening a pathway to apply traditionally stiff and brittle materials in high‐deformation applications such as biomedical implants.

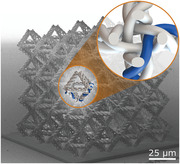

Materials with hierarchy are abundant in nature, and their combination of structural hierarchy at different length scales gives rise to bulk properties unattainable independently by their constituent materials.^[^ ^1^ ^]^ While architectural hierarchy has been shown to enhance damage tolerance such as in mantis shrimp claw and elk antler bone,^[^ ^2^, ^3^ ^]^ hierarchical structures in bone and nacre lead to fracture toughness values that are higher than their respective building blocks.^[^ ^1^, ^4^, ^5^ ^]^ Advances in fabrication methods have enabled the creation of synthetic materials with similar structural hierarchy down to the micro‐ or nanometer scale, leading to desirable properties such as extreme resilience and high deformability.^[^ ^6^, ^7^, ^8^ ^]^ Beyond static mechanical properties, hierarchical architected materials have also opened up new material properties such as tunable ultrasonic band gaps,^[^ ^9^, ^10^ ^]^ ultralow or tunable thermal response,^[^ ^11^, ^12^, ^13^ ^]^ and impact resistance.^[^ ^14^ ^]^

Despite this broad property space enabled by structural hierarchy, most three‐dimensional (3D) synthetic architected materials have drawn from beam‐and‐junction‐based design principles.^[^ ^6^, ^15^, ^16^, ^17^ ^]^ These types of designs have been studied extensively both experimentally and computationally,^[^ ^18^, ^19^, ^20^ ^]^ and are characterized for their high stiffness‐ or strength‐to‐weight ratios. Even with enabling these properties, the presence of nodes has been identified to be detrimental in some cases, particularly at higher relative densities (i.e., fill fractions) where nominally stretching‐dominated architectures (as defined by the kinematic rigidity of the unit cells)^[^ ^21^ ^]^ exhibit a less‐desirable bending‐dominated response.^[^ ^18^, ^22^ ^]^ These materials are appealing in a number of applications where maximal linear mechanical properties are required, but lose their optimality in large deformations due to their failure mechanisms and constrained kinematics. In particular, few architected materials made of materials stiffer than elastomers have been reported to withstand deformations greater than 20% strain,^[^ ^23^, ^24^ ^]^ and most of them fail catastrophically or accumulate significant damage.^[^ ^11^, ^25^, ^26^, ^27^ ^]^ The geometries in these materials lead to stress concentration at junctions or nodes where damage nucleates,^[^ ^11^, ^28^, ^29^ ^]^ commonly resulting in significantly weaker or compliant responses after the initial deformation. While higher stiffness‐ and strength‐to‐weight ratios can be achieved by choosing closed‐cell, plate‐based designs over beam‐and‐junction‐based designs, the deformability of such architected materials is still limited.^[^ ^30^, ^31^, ^32^ ^]^ As an alternative, architected materials that lack junctions or nodes, such as triply periodic minimal surface and stochastic spinodal shell designs,^[^ ^24^, ^33^, ^34^, ^35^ ^]^ more evenly distribute stresses throughout their components but have not yet enabled repeatable large deformations without significant degradation except for designs with very low material fill fraction.^[^ ^36^ ^]^ Wire‐woven architected materials have recently been reported to have desirable energy absorption capabilities and buckling suppression,^[^ ^37^, ^38^ ^]^ presenting a potential approach to enable repeatable deformability, but have lacked the introduction of hierarchy to further enhance these properties.

Here, we present a new type of hierarchy in lattice architectures that consists of weaving structural components to assemble an effective beam, replacing the classical monolithic‐ or hollow‐beam design (**Figure** **1**).^[^ ^6^, ^17^ ^]^ Using these hierarchical woven beams as building blocks in a periodic unit cell, of the same type as their monolithic and hollow counterparts, results in woven lattice architectures. At the effective beam junctions (i.e., nodes) of woven lattices, fibers are not combined to form monolithic junctions but are instead interwoven into adjacent beams. Since these woven lattice architectures open up many previously unexplored design spaces in fiber geometry and connectivity, we fixed certain design parameters to allow a systematic study of their mechanical response. For instance, each woven beam of length *L* is designed to consist of three fibers, which are woven in the form of a full helical rotation with pitch *λ* = *L*, that extend beyond a given beam to also make up the three closest neighboring woven beams (Figure 1a). In this work, we design, fabricate, and compare the mechanical response of woven and monolithic lattices of two common unit‐cell configurations: i) the kinematically rigid octahedron, and ii) the non‐rigid diamond unit cell. To understand the fundamental effect of fiber geometry on the mechanical properties, we also perform numerical studies on the axial and bending stiffnesses of woven beams with varying geometric parameters while maintaining constant relative density (i.e., fill fraction).

**Figure 1:**
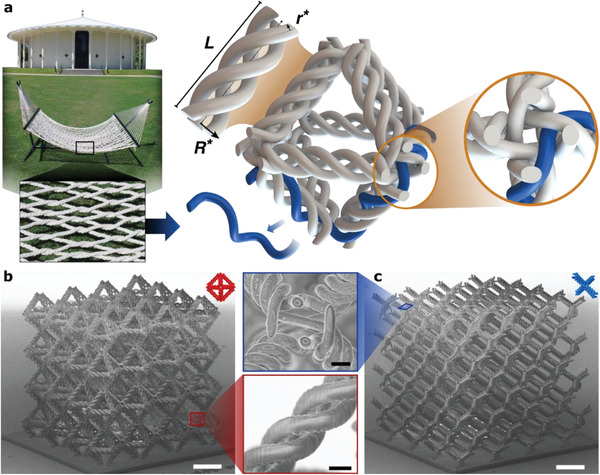
Design and realization of micro‐woven lattices, whose beam and junction geometries are analogous to woven ropes. a) Computer‐aided design (CAD) images of an octahedron unit cell of a woven lattice showing non‐intersecting fibers that form its beams and junctions. Scanning electron microscope (SEM) images of the b) woven octahedron and c) woven diamond lattices ($\bar{\rho }\approx$ 5%) with their corresponding CAD representations, and detailed views of a woven beam and a junction in a woven diamond lattice. Scale bars in white correspond to 20 µm, and black bars in insets are 2 µm.

The samples were fabricated at the microscale using two‐photon lithography out of IP‐Dip photoresist (Nanoscribe GmbH), which enabled the creation of intricate fiber geometries with sub‐micron radii r*. These samples consisted of 4 × 4 × 4 tessellations of 30 µm unit cells, with intended relative densities $\bar{\rho }$ of 3.3% to 5% for diamond lattices and 5% to 8% for octahedron lattices. While the unit cell size was held constant, the fiber radius r* and the beam radius R* were designed to vary from 0.6 to 0.9 µm and 1.85 to 2.8 µm, respectively, to attain different relative densities. To enable tension‐to‐compression nanomechanical experiments, custom grips were fabricated on top of the woven lattices while a plate was used at the base of the samples to promote adhesion to the Si substrate. For comparison purposes, we also fabricated monolithic octahedron and diamond samples of identical tessellations and relative densities to the woven samples, with monolithic beam radii ranging from 1.2 to 1.8 µm.

We performed in situ uniaxial tension experiments on at least three samples per configuration, which showcased an enhanced extensibility of woven architectures compared to their monolithic beam‐and‐junction‐based counterparts. For this comparative study, we chose the stiffest experimentally viable set of parameters for a woven lattice of a given $\bar{\rho }$ and unit cell configuration. **Figure** **2**a,b show the tensile responses up to failure for woven and monolithic lattices at a relative density of $\bar{\rho }\approx$ 5%, with insets showing the progression of deformation along the experiments. These experiments show that woven lattices in tension attain a ∼70%–120% increase in strain at the point of ultimate failure compared to monolithic lattices, for both octahedron and diamond geometries, with the non‐rigid diamond configuration exhibiting the largest average extensibility of 64% strain. The architectural redundancy from having three fibers in an effective beam—as opposed to a single continuous body—is also shown to allow woven lattices to elongate further upon ultimate failure instead of completely rupturing (Figure 2aIV,bIV and Figure S1, Supporting Information). In addition, smooth structural reconfiguration through uncoiling and reorientation of fibers in the woven beams and junctions enabled enhanced elongation (Figure 2aI–IV,bI–IV). Woven lattices with octahedron unit cells (Figure 2, bold red) reached ultimate tensile failure strain $\varepsilon_{t,\;f}$ of 40.6 ± 1.0%, and ultimate tensile strength $\sigma_{t,\;f}$ of 515 ± 52 kPa, while woven lattices with diamond unit cells (Figure 2, bold blue) had $\varepsilon_{t,\;f}$ = 64 ± 9% and $\sigma_{t,\;f}$ = 277 ± 4 kPa. In comparison, monolithic samples resulted in $\varepsilon_{t,\;f}$ = 23.4 ± 0.7% and $\sigma_{t,\;f}$ = 1113 ± 206 kPa for the octahedron configuration, and $\varepsilon_{t,\;f}$ = 28.9 ± 0.6% and $\sigma_{t,\;f}$ = 759 ± 72 kPa for the diamond configuration.

**Figure 2:**
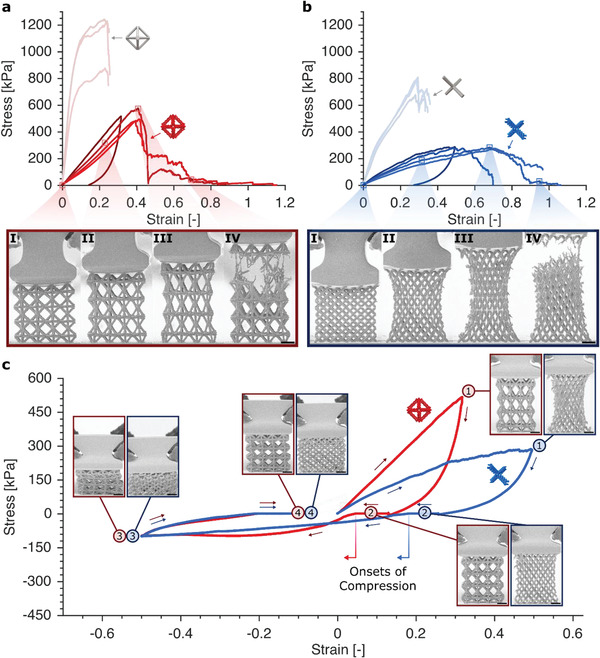
Tension experiments of woven and monolithic lattices ($\bar{\rho }\approx$ 5%), and combined tension‐to‐compression experiments of woven lattices. Tensile responses of woven and monolithic lattices with a) rigid, octahedron and b) non‐rigid, diamond unit cell configurations loaded up to failure. Red and blue data points correspond to octahedron and diamond configurations, respectively. Faint colors indicate monolithic lattices, and bold colors are for woven lattices, with the boldest showing the tensile portion of the tension‐to‐compression data in (c). Insets show still frames from two tensile experiments (one for each woven architecture) corresponding to locations in the graphs. c) Tension‐to‐compression experiments of woven octahedron and diamond lattices with insets for each labeled region. Scale bars correspond to 25 µm.

To further showcase the versatility of these woven lattices, we performed continuous tension‐to‐compression experiments corresponding to absolute strain changes Δε of up to 118% without sample failure. Figure 2c presents a comparison between the woven octahedron and diamond geometries (both of $\bar{\rho }\approx$ 5%) undergoing quasi‐static tension‐to‐compression deformation (corresponding movies are shown in Figures S2 and S3, Supporting Information). Regions of maximum tension and maximum compression are designated by regions 1 and 3 in Figure 2c, respectively. At maximum tensile strains (region 1, Figure 2c) of 32% and 50% in the octahedron and diamond geometries, respectively—beyond $\varepsilon_{t,\;f}$ of each geometry's corresponding monolithic lattices—woven beams were observed to accommodate for the Poisson effect by reorienting and uncoiling in the direction of applied load. Plateaus in regions 2 and 4 correspond to transitions between strain type (i.e., tension or compression), where the indenter tip was not in contact with the top or bottom edges of the grip. Upon full tensile unloading (end of region 2), the viscoelastic response of IP‐Dip accelerated the onset of compression (see Figure S4, Supporting Information), which is indicated by an arrow corresponding to each woven lattice geometry. Smooth reorientation of beams was again observed at the maximum compression point (region 3), accommodating for deformation without significant permanent damage. As in the tensile unloading region, the compressive unloading region (region 4) came with a concomitant viscoelastic response.

We probed the response of woven lattices upon repeated deformation by performing additional cyclic compression experiments to 35% strain while comparing them to monolithic samples of both octahedron and diamond geometries. The response of each monolithic configuration was characterized by catastrophic failure events or significant plastic buckling in the first cycle, resulting in drastically different subsequent cycles due to accumulated damage (**Figure** **3**a,b). In contrast, the woven octahedron and diamond samples had more self‐similar loading cycles despite a pronounced viscoelastic effect, exhibiting no beam or fiber failure after ten cycles. To quantify their mechanical resilience, we calculated the dissipated energy density per *i*‐th compression, $\Delta U_{i}$, as the area enclosed by each stress (σ) versus strain (ε) cycle,

(1) $$\begin{array}{c} \Delta U_{i}=\oint \sigma \;d\varepsilon \; \end{array}$$

and report the first‐cycle‐normalized quantities $\Pi_{i}$, defined as

(2) $$\begin{array}{c} \Pi_{i}=\frac{\Delta U_{i}}{\Delta U_{1}}. \end{array}$$

**Figure 3:**
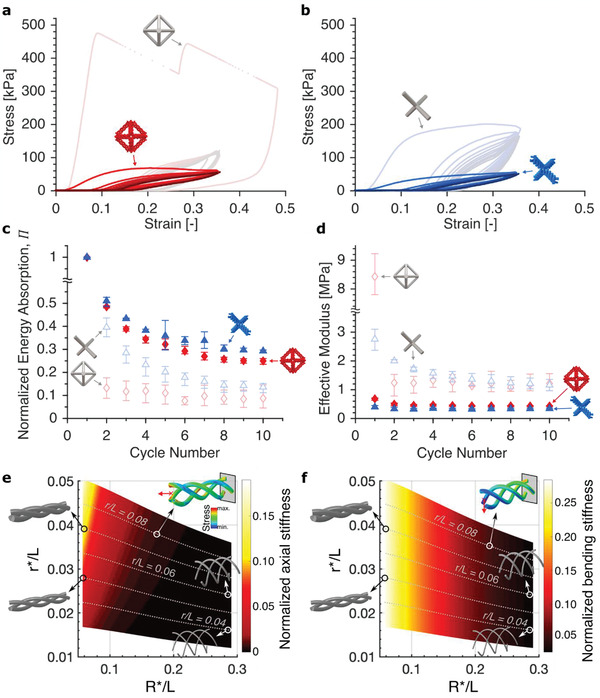
Compression responses and numerical studies of woven and monolithic architectures. Representative mechanical responses from cyclic compression of a) octahedron and b) diamond lattices ($\bar{\rho }\approx$ 5%) of both woven and monolithic architectures for ten cycles. Darker color represents a later cycle. c) Normalized energy absorption of each lattice architecture as a function of load‐unload cycle showing higher resiliency for woven lattices. d) Effective modulus of each lattice architecture as a function of load‐unload cycle. Error bars indicate the extrema of the data sets. Numerical studies on e) axial and f) bending stiffnesses of woven beams as functions of woven beam dimensionless geometric parameters r*/L and R*/L. The dashed grey lines represent constant‐volume paths corresponding to the volume of a given monolithic strut with slenderness *r/L*. Woven‐beam stiffnesses were normalized by those of the corresponding equal‐volume monolithic strut, showing the ability of woven architecture to reach higher compliance and decouple variations in axial and bending stiffnesses.

Figure 3c shows that by the end of the tenth cycle, the normalized energy absorption values for woven lattices with $\bar{\rho }\approx$ 5% settled at 24%–30%, values ∼2–3 times higher than those for monolithic lattices of the same unit cell configurations. Although for woven lattices these normalized energy absorption values correspond to less than 3 kJ m^−3^ in absolute absorbed energy densities *W*_abs_ and less than half the *W*_abs_ for monolithic lattices (Figure S5, Supporting Information), the average *W*_abs_ for woven lattices in tension‐to‐failure experiments differs by no more than 10%–35% of the average *W*_abs_ for monolithic lattices (Figure S6, Supporting Information). In addition, the evolution of effective Young's modulus per cycle in Figure 3d shows that the effective moduli for woven configurations settled after approximately three cycles (see Figures S7–S9, Supporting Information for cyclic responses in tension and Figure S10, Supporting Information for cyclic compression of lattices with different relative densities). Higher tenth‐cycle energy values, and this early settling of the effective Young's modulus, indicate that woven lattices accrued less damage during cyclic loading. These cyclic compressions also show that octahedron lattices generally had lower normalized energy values compared to diamond lattices for both woven and monolithic configurations, despite having first‐cycle absolute energy absorption values that were 39% and 135% higher for woven and monolithic configurations, respectively.

Besides improving the extensibility and minimizing the mechanical degradation of the architectures, woven hierarchy enables linear mechanical properties that are unachievable using monolithic geometries, such as those desired in materials for flexible electronics or wearable devices.^[^ ^39^, ^40^ ^]^ We explored the range of properties enabled by this type of hierarchy by performing a numerical study on the woven‐beam component that makes up these architectures. Using the finite element method, we computed maps of axial and bending stiffnesses of a cantilever woven beam as a function of i) the ratio between the effective beam radius and beam length, R*/*L*, and ii) the ratio between the fiber radius and beam length, r*/*L*. For comparison to its monolithic counterpart, the computed woven stiffnesses were normalized by the corresponding stiffnesses of a monolithic cantilever beam with the same material volume and length (Figure 3e,f). The stress contours in these simulations indicate that woven beams are inherently bending‐dominated, implying that unit cell architecture and kinematic rigidity should play a minor role in the linear mechanical response. This explains the similarity in modulus and energy absorption for the woven octahedron and diamond configurations, since both are bending‐dominated, opposite of what is expected and observed for rigid and non‐rigid monolithic configurations. In addition to introducing bending deformation, woven hierarchy also allows the transition from catastrophic to non‐catastrophic failure in the octahedron geometry by mitigating stress concentration at nodes. Since failure stresses are not reached at nodes anymore, the added compliance of woven nodes facilitates beam alignment in the direction of the applied load, which together with uncoiling of helical fibers enables the observed extreme extensibility. The stiffness maps also exhibit a markedly different evolution of stretching and bending stiffnesses as functions of r*/*L* and R*/*L*, where normalized stretching stiffness increases while normalized bending stiffness stays relatively constant as r*/*L* rises on a given R*/*L*. The decoupling of the beam's axial and bending stiffnesses via woven architecture opens up new ways to prescribe directional compliance in an architected material that are inaccessible to classical beam‐and‐junction‐based designs.^[^ ^41^ ^]^

The ability to increase the compliance and deformability of a given material is intriguing as it opens up possibilities to incorporate stiff and brittle materials in high‐deformation and load‐sensitive applications.^[^ ^39^ ^]^ Early successes have been shown in the field of stretchable electronics, where researchers have incorporated thin and wavy planar architectures to allow a considerable amount of stretching, bending, and twisting in devices containing brittle silicon. Yet such pronounced deformability is not easily attainable for thick materials of arbitrary shapes; increasing the tensile failure strain of a given material via 3D architecture has proven to be a non‐trivial task, as evidenced by recent works on metallic and polymeric bending‐dominated foams which counterintuitively achieved a lower tensile failure strain than their constituent materials.^[^ ^42^ ^]^ As an alternative to the stochastic structures present in foams, woven hierarchy provides one method to achieve extensibility beyond that of the constituent materials, in addition to an already superior extensibility compared to other periodic architected materials (**Figure** **4**a,b).^[^ ^23^, ^29^, ^43^, ^44^ ^]^ To allow comparison across architectures and materials (Figure 4b), we define a normalized tensile stress at failure ${\bar{\sigma }}_{t,\;f}$ as the tensile stress at failure $\sigma_{t,\;f}$ normalized by the relative density of the architected material and the tensile strength of the constituent material $\sigma_{s}$, and we simply define a normalized tensile strain at failure ${\bar{\varepsilon }}_{t,\;f}$ as the tensile strain at failure $\varepsilon_{t,\;f}$ normalized by the maximum tensile strain of the constituent material $\varepsilon_{s}$. After determining the $\sigma_{s}$ and $\varepsilon_{s}$ of the constitutive material in our lattices by testing IP‐Dip pillars in tension (see Figures S11 and S12, Supporting Information), we arrived at normalized failure strength and strain values for comparison to other reported architected materials.^[^ ^43^, ^45^, ^46^, ^47^, ^48^ ^]^ An initial comparison between the samples in our study shows that the tensile failure strain $\varepsilon_{t,\;f}$ of IP‐Dip monolithic octahedron and diamond lattices were lower than that of IP‐Dip pillars, whereas woven IP‐Dip lattices were able to deform past the tensile failure strain of IP‐Dip, with the diamond configuration deforming up to 73% strain. Compared to the most extensible non‐elastomeric architected material in literature, IP‐Dip lattices in this work attained 5–25 times higher tensile strength while also outperforming its extensibility by more than 5% and up to 221%. Beyond merely joining a special class of architected materials that can elongate farther than their constituent materials, the woven lattices presented in this work achieve this feat while providing an alternate and wider design parameter space via their added compliance and the tunability of their effective beams’ axial and bending stiffnesses; an even higher elongation of woven lattices with equal mass could potentially be achieved by opting for larger fiber arc lengths and more separation between each fiber.

**Figure 4:**
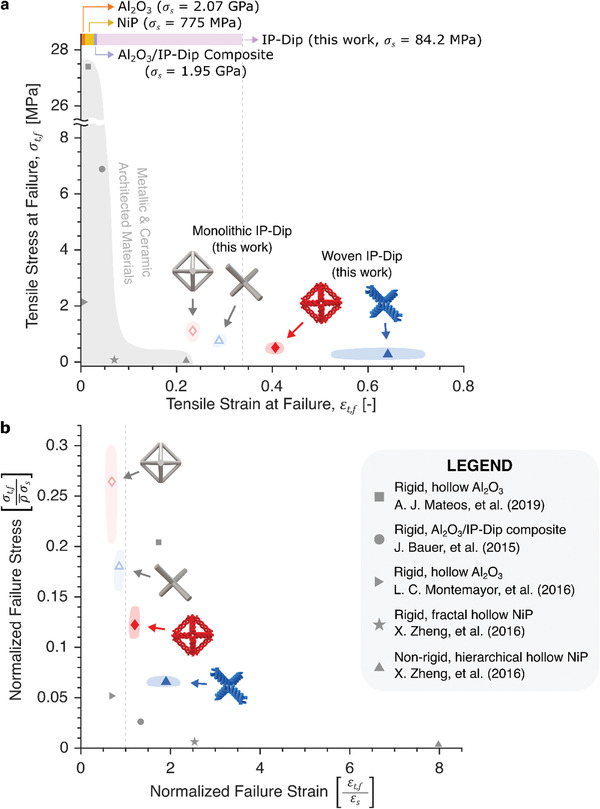
Performance comparison between lattices in this work and other architected materials in literature. a) Tensile stress at failure $\sigma_{t,\;f}$ versus elongation to failure $\varepsilon_{t,\;f}$ of woven and monolithic IP‐Dip lattices in Figure 2a,b compared to other architected materials. The bars at the top of the graph show the maximum tensile strains $\varepsilon_{s}$ of the relevant constituent materials. b) Normalized tensile stress at failure ${\bar{\sigma }}_{t,\;f}$ versus normalized tensile strain at failure ${\bar{\varepsilon }}_{t,\;f}$ of architected materials in (a). The failure strain $\varepsilon_{t,\;f}$ for ref. [29] was measured from video of experiment. The constituent material strength $\sigma_{s}$ for Al_2_O_3_ and NiP were calculated by taking the average of the values reported in refs. ^[^ ^45^ ^]^ and ^[^ ^47^ ^]^, respectively. An estimate in ref. [48] was used for $\varepsilon_{s}$ of Al_2_O_3_, while $\varepsilon_{s}$ of NiP was taken from the reported value in ref. [46]. For the Al_2_O_3_/IP‐Dip composite, the $\varepsilon_{s}$ was calculated by dividing the average alumina fracture stress with the average elastic modulus of the alumina reported in ref. [43] along with the $\sigma_{s}$.

In summary, we introduced a new type of hierarchical architecture for periodic architected materials, where fibers are interwoven to construct effective beams and thus stretching the limits of deformability and compliance of the material while still being able to preserve the conventional connectivity of well‐known periodic unit cells. These woven lattices exhibited smooth reconfiguration of woven microfibers in the effective beams and junctions, giving rise to exceptional tensile failure strain and less accrued damage while preventing catastrophic failure under compression. Structural redundancy, from having multiple fibers in an effective beam, also showed the potential for woven microlattices to elongate further after ultimate tensile failure points were reached. Through finite element models, maps of axial and bending stiffnesses of woven beams with varying geometric parameters present new design spaces for periodic architected materials with programmed compliance. The demonstrated capabilities of woven hierarchical architecture make it a great candidate for materials in high‐deformation or load‐sensitive systems, while the contact interaction between fibers in an effective beam offers a building block for materials with complex nonlinear mechanics such as selective damping.

## Experimental Section

##### Additive Manufacturing via Two‐Photon Lithography

All samples were fabricated out of IP‐Dip photoresist via two‐photon lithography using a commercially available system (Photonic Professional GT, Nanoscribe GmbH). Each sample was written on a Si substrate using laser power and scan speed of 15 mW and 10 mm s^−1^, respectively. All lattices were printed with equal hatching and slicing distance of 0.1 µm, with the exception of monolithic octahedron lattices that were printed with hatching and slicing distances of 0.2 and 0.1 µm, respectively; for both woven and monolithic lattices, a contour line was printed on the perimeter of each printed cross‐section. All IP‐Dip pillars were printed with equal hatching and slicing distance of 0.2 µm and without contour line. For tension and tension‐to‐compression samples, the Si substrates were silanized prior to writing to improve adhesion between the samples and the chips. Critical point drying was performed on written samples using Autosamdri 931 (Tousimis). To ensure that the tested lattice samples had relative densities close to the intended design, radii of selected fibers were measured from the top and/or the side of each sample. Fiber cross‐sectional areas were then estimated using the radii measurements and compared to the cross‐sectional area in the design.

##### In Situ Mechanical Experiments

Quasi‐static uniaxial tension and tension‐to‐compression experiments were performed using a custom‐made tension tip attached to a nanoindenter (InSEM, Nanomechanics Inc.) installed in an SEM (FEI Quanta 200F) to enable in situ imaging of the experiments. In situ quasi‐static compression experiments were performed using a compression tip on the same nanoindenter, and cyclic ex situ compression experiments were performed in a G200 XP Nanoindenter (Agilent Technologies) up to ≈35% strain. For each architecture (woven vs. monolithic) and unit cell configuration, three samples were tested in tension up to failure and at least three samples were tested under cyclic compression. Normalized energy absorption and effective modulus data from compression cycles that did not go all the way to 35% strain were omitted. All lattice samples were loaded at a strain rate of 3 × 10^−4^ s^−1^, and all pillars were loaded at a displacement rate of 30 nm s^−1^, which falls around the machine displacement‐limit and corresponds to a strain rate of 4.3 × 10^−4^ s^−1^. Stress is calculated using $\sigma \;=\frac{P}{A}$, where *P* and *A* are load and the initial cross‐sectional area of the lattice perpendicular to vertical loading direction, respectively. Engineering strain is calculated by normalizing vertical displacement by the gauge length. In tension and tension‐to‐compression experiments, measured vertical displacements were corrected to account for the compliance of the IP‐Dip grip heads and base (see Figures S13 and S14 and Equation S1, Supporting Information). The absolute absorbed energy density *W*_abs_ is calculated as the area inside the stress‐strain curve, which equals to Equation (1) for cyclic experiments.

##### Statistical Analysis

Explicit outliers in cyclic compression data were removed. The toe region of the stress‐strain curve, which indicates the measured initial deformation before full contact was established between the sample and the indenter tip, was removed from each data set except for cyclic compression data. Unless otherwise noted, experimental lattice data came from at least three different samples and are presented as mean ± standard deviation. Statistical analysis was carried out using MATLAB.

## Conflict of Interest

The authors declare no conflict of interest.

## Author Contributions

W.P.M. fabricated the samples, conducted the experiments, and compiled the experimental data. W.P.M., A.J.M., J.R.G., and C.M.P. conceived and designed the experiments. R.M.F. conceived the original idea of woven lattices. C.M.P. performed the finite element simulations. W.P.M., J.R.G., and C.M.P. analyzed the data and discussed the findings. W.P.M. and C.M.P. wrote the paper.

## Supporting information

Supporting Information

Supplemental Figure 2

Supplemental Figure 3

Supplemental Figure 8

Supplemental Figure 9

Supplemental Figure 12
