# Supplementary material for: Pushing and Pulling on Ropes: Hierarchical Woven Materials
Source: Adv Sci (Weinh). 2020 Aug 24;7(20):2001271. doi: 10.1002/advs.202001271 (PMC7578876; doi:10.1002/advs.202001271)
Supplement: Supplementary file 1 — Supporting Information [file ADVS-7-2001271-s001.pdf]

Copyright WILEY-VCH Verlag GmbH & Co. KGaA, 69469 Weinheim, Germany, 2018.

Supporting Information

Pushing and Pulling on Ropes: Hierarchical Woven Materials

*Widianto P. Moestopo, Arturo J. Mateos, Ritchie M. Fuller, Julia R. Greer, and Carlos M. Portela**

W.P. Moestopo, Dr. A.J. Mateos, Prof. J.R. Greer, Prof. C.M. Portela
Division of Engineering and Applied Science, California Institute of Technology, CA 91125 USA

R.M. Fuller
Independent Artist, USA

Prof. C.M. Portela
Department of Mechanical Engineering, Massachusetts Institute of Technology, Cambridge, MA 02139, USA
E-mail: cportela@mit.edu

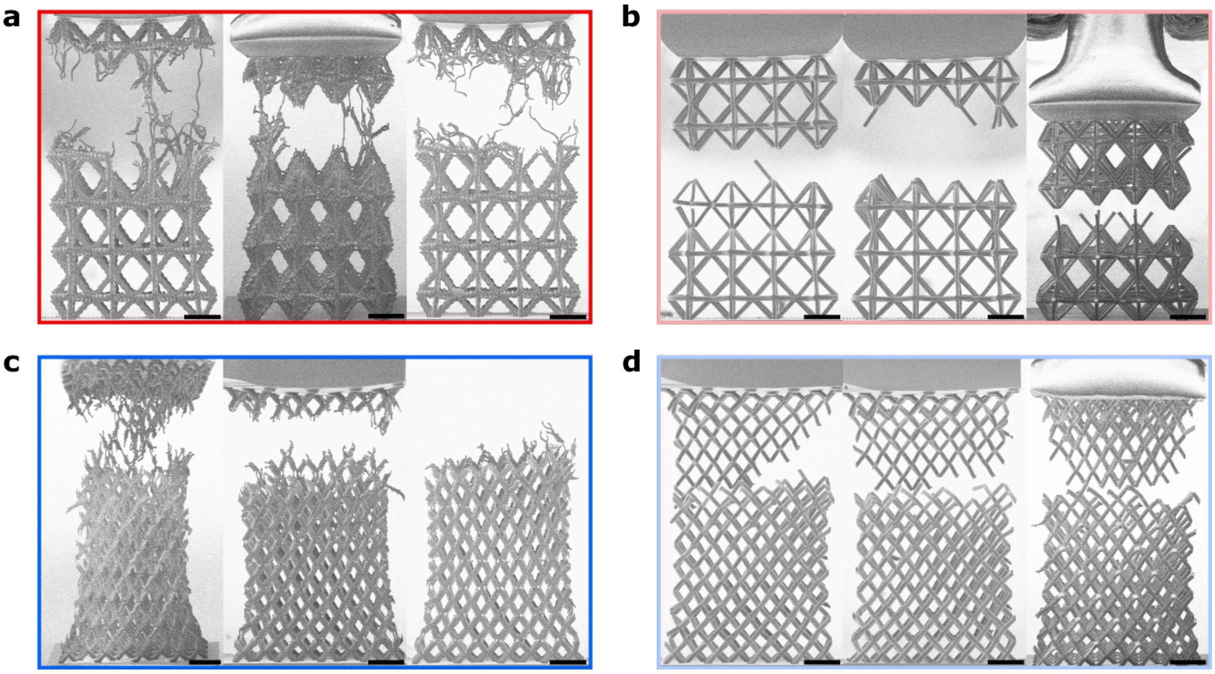

Figure S1. Fractographs of woven and monolithic lattices ($\bar{\rho} \approx 5\%$) loaded in tension: (a) woven octahedron, (b) monolithic octahedron, (c) woven diamond, and (d) monolithic diamond lattices. Scale bars are 25 μm.

Figure S2. Movie comparing the tensile and compressive behaviors of woven octahedron lattice from Figure 2c (shown in red) and monolithic octahedron lattices (shown in gray) for $\bar{\rho} \approx 5\%$ at x150 playback speed. The still image of monolithic octahedron lattice at the end of the video shows the image of the lattice after two cycles of compression up to 70% strain.

Figure S3. Movie displaying the tension-compression experiment of a woven diamond lattice ($\bar{\rho} \approx 5\%$) shown in Figure 2c at x150 playback speed.

Determination of Quasi-Static Strain Rate

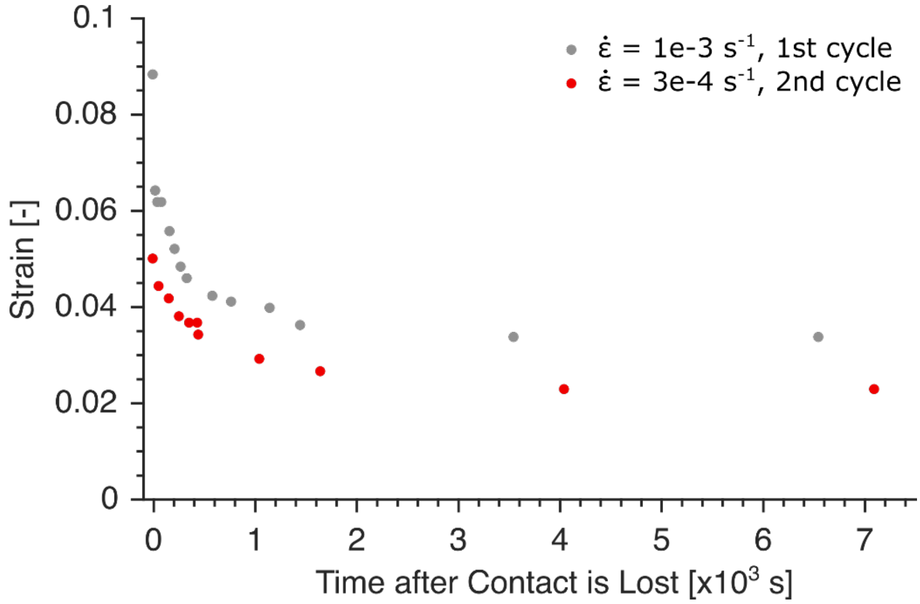

Figure S4. Compressive strain vs. time data showing viscoelastic creep phenomena of octahedron IP-Dip woven lattices ($\bar{\rho} \approx 5$ %) under two different strain rates once contact was lost between the indenter tip and the corresponding sample during the unloading portion of a compression up to 30% strain.

To determine the acceptable strain rate for our quasi-static experiments, we performed compression tests using the strain rate that is commonly found in literature (1 x 10^{-3} s^{-1}) and the lowest achievable strain rate for our testing set-up (3 x 10^{-4} s^{-1}). As shown in Figure S1, the lower strain rate led to a smaller strain lag between the indenter and the sample when they lost contact (~1% less strain than with the larger strain rate), and it settled at a lower strain. Therefore, we concluded that all experiments should be conducted at a strain rate that was as close to 3 x 10^{-4} s^{-1} as possible.

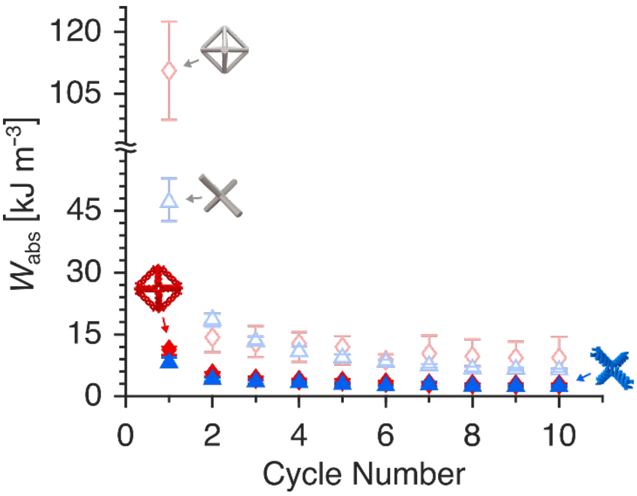

Figure S5. Absolute absorbed energy densities (W_{abs}) of lattices in Figure 3a-d as a function of load-unload cycle. Error bars indicate the extrema of the data sets.

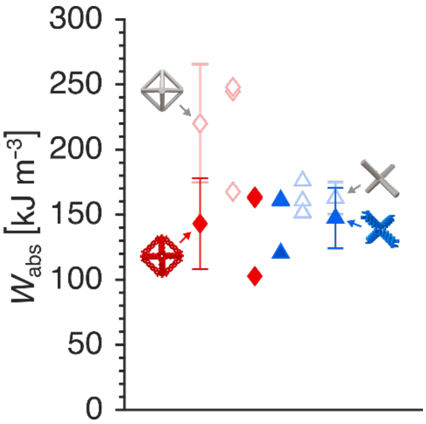

Figure S6. Absolute absorbed energy densities (W_{abs}) of lattices loaded in tension up to failure as shown in Figure 2a,b. Error bars represent the standard deviation of the data sets.

Cyclic Tension Experiments of Woven Lattices

To further explore the energy absorption capability of woven lattices in tension, we performed additional cyclic tensile experiments on woven octahedron and diamond lattices up to five cycles (Figure S7). We tested one woven octahedron lattice ($\bar{\rho} \approx 5\%$) up to 26% strain and one woven diamond lattice of the same relative density up to 40% strain, which corresponded to ~62-65% of each lattice's average ultimate failure strain. Lattice geometry was observed to play a minor role since the added compliance in the diamond lattice was offset by an increased extensibility compared to the octahedron lattice, resulting in almost identical absolute energy absorption values. Direct comparison of the energy densities in Figure S5 (compression) and Figure S7c (tension) shows that the woven octahedron lattice can attain higher absorbed energy densities in tension throughout 5 cycles, despite being deformed to lower strains.

Unlike the trend seen in Figure 3d, Figure S7b shows that for both lattices, there was an increase in stiffness in subsequent cycles. In the corresponding movies shown in Figures S8 and S9, smooth reorientation of the beams can be observed during loading, but the beams had not fully returned to their initial configuration before the next cycle began. Since in the beginning of the subsequent cycle the beams were more aligned toward the loading direction compared to the previous cycle, the calculated effective modulus for the subsequent cycle understandably became higher than the calculated value from the previous cycle.

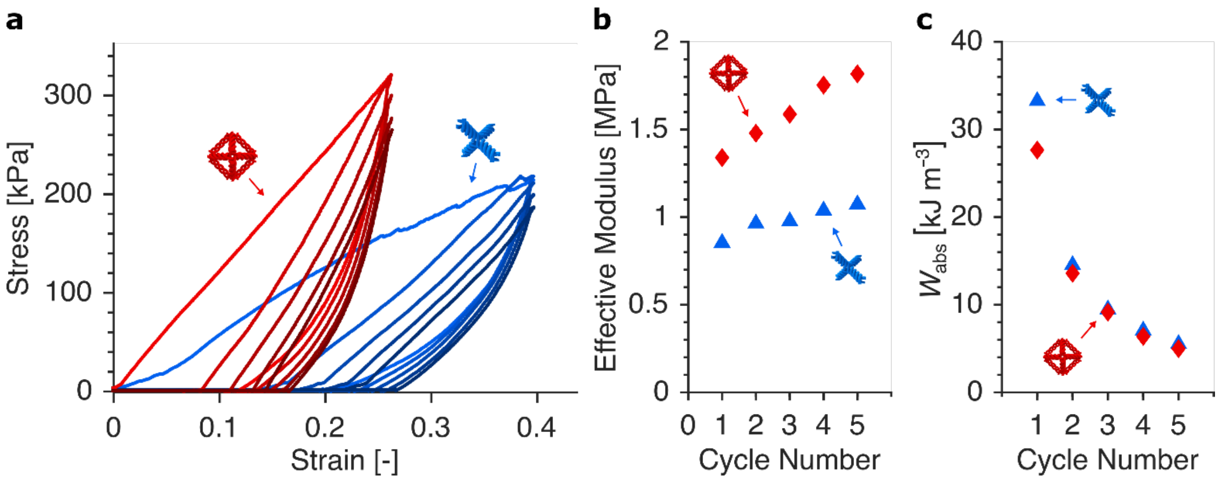

Figure S7. Cyclic tension responses of woven architectures. a) Mechanical responses from cyclically loading a woven octahedron and a woven diamond lattice ($\bar{\rho} \approx 5\%$) up to five cycles. Darker color represents a later cycle. b) Effective modulus of each lattice as a function of load-unload cycle. c) Absolute absorbed energy density (W_{abs}) of each lattice as a function of load-unload cycle.

Figure S8. Movie of the first three cycles of cyclic tensile testing of a woven octahedron lattice ($\bar{\rho} \approx 5\%$) shown in Figure S7 at x500 playback speed.

Figure S9. Movie of the first three cycles of cyclic tensile testing of a woven diamond lattice ($\bar{\rho} \approx 5\%$) shown in Figure S7 at x500 playback speed.

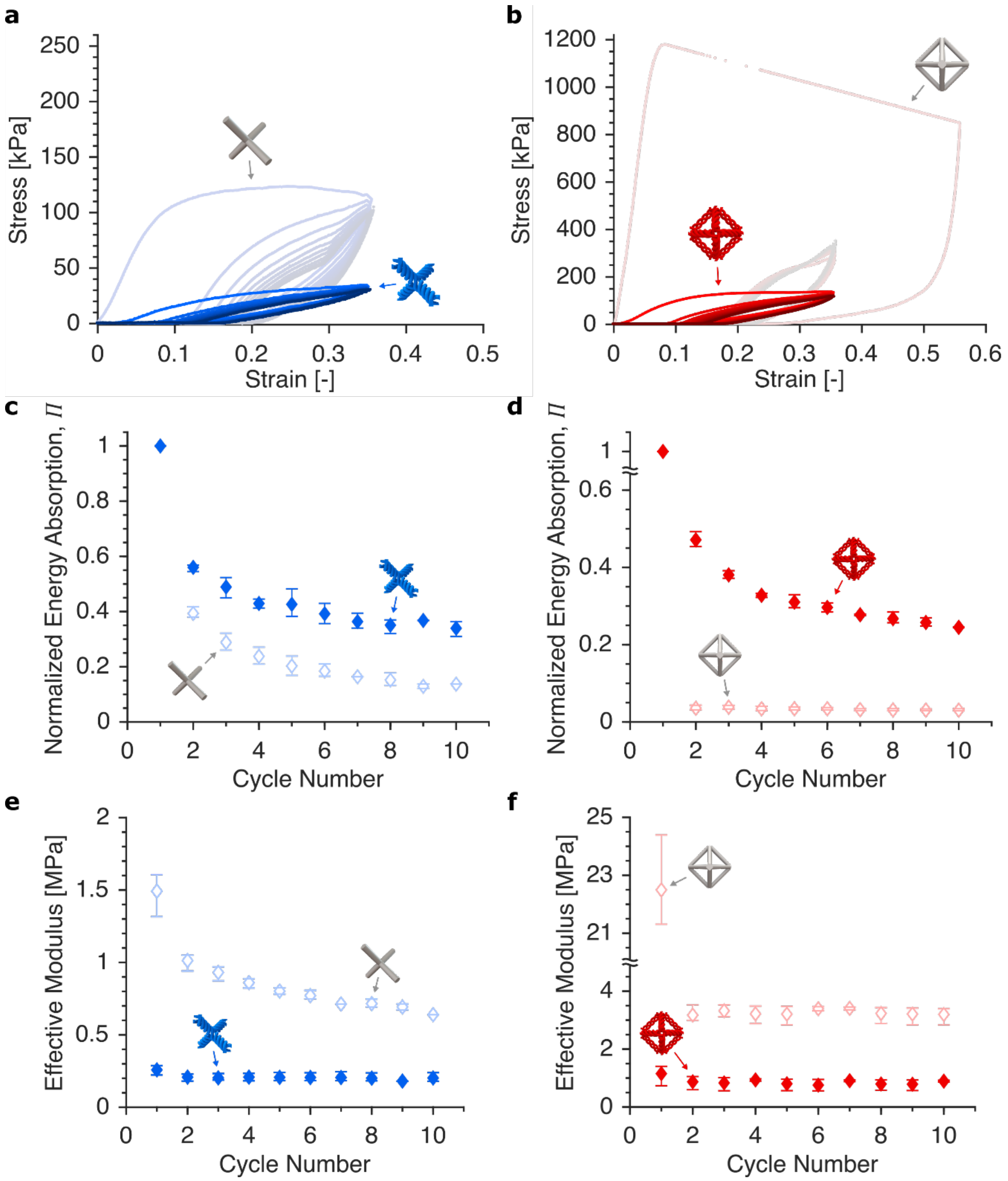

Figure S10. Representative cyclic compression responses and the calculated mechanical properties of woven and monolithic (a, c, e) diamond lattices with $\bar{\rho} \approx 3.3$ %, and (b, d, f) octahedron lattices with $\bar{\rho} \approx 8$ %. Error bars indicate the extrema of the data sets. Faint colors indicate monolithic lattices, and bold colors are for woven lattices. Darker color in a,b indicates a later cycle.

Properties of IP-Dip in Tension

Tension experiments up to failure were performed on three IP-Dip pillars with average sample height of 69.90 ± 0.15 µm and average sample diameter of 13.43 ± 0.03 µm (see Figure S11 and movie in Figure S12). As with lattice experiments, the displacement data from the experiments were corrected accordingly by taking into account the compliance of the IP-Dip grip heads and bases. We observed yielding behaviors and obtained the following material properties: tensile stress at failure, $\sigma_{t,f}$ = 84.2 ± 4.1 MPa, tensile strain at failure, $\varepsilon_{t,f}$ = 33.7 ± 2.0%, Young's Modulus = 1.49 ± 0.34 GPa, and yield stress, σ_{yield} = 26.8 ± 5.9 MPa, which was determined via 0.2% yield offset of the engineering stress-strain curves.

We now compare the mechanical properties of our IP-Dip pillars to reported values of pillars with similar dimensions and predicted "degree of conversion" (DC), which is a measure of the amount of polymer chain cross-linking as described in Reference 1. Compared to a pillar tested in tension with DC of ~23%, our pillars had comparable Young's Modulus (1.49 GPa vs 1.5 GPa) and lower $\sigma_{t,f}$ (84.2 MPa vs 115 MPa), which may have resulted from having a larger hatching distance (i.e. distance between printed lines in a layer) during the printing of our pillars. When compared to pillars of similar printing parameters that were tested in compression, our pillars had lower σ_{yield} and Young's Modulus (72 MPa and 2.68 GPa, respectively).[2]

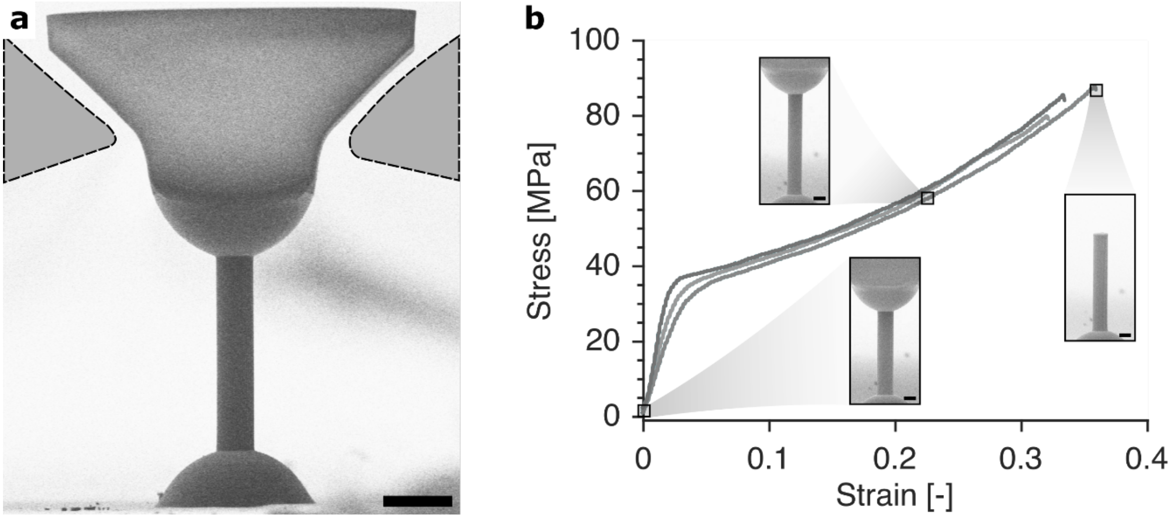

Figure S11. Tensile testing of IP-Dip pillars. a) Micrograph showing a pillar with an attached custom grip for tensile testing. b) Mechanical responses of pillars tested to determine the mechanical properties of IP-Dip in this study. Scale bars in (a) and (b) are 25 µm and 10 µm, respectively.

Figure S12. Movie showing an IP-Dip pillar loaded in tension up to failure at x100 playback speed.

Compliance Correction in Tension and Tension-to-Compression Experiments

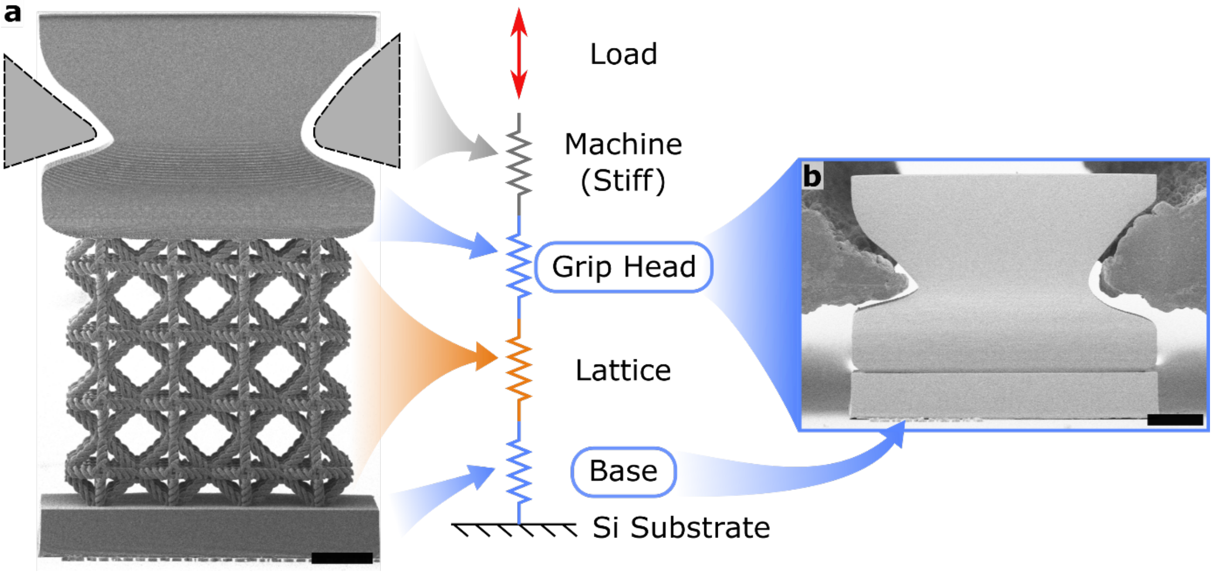

Figure S13. (a) Mechanical representation of the experimental set-up for in situ tension and tension-to-compression experiments. (b) Testing set-up to measure the stiffness of the IP-Dip grip head and base. The grip head shown in both images was designed for tension-to-compression experiment of octahedron samples. Scale bars are 25 µm.

Due to less-than-optimal video quality that has been deemed inadequate to produce accurate digital image correlation, we have chosen to methodically perform a rigorous compliance correction procedure to present accurate tensile data. The custom set-up for tension and tension-to-compression experiments requires the fabrication of IP-Dip grip head via two-photon lithography directly after the writing of each sample. An IP-Dip base was also fabricated underneath each lattice sample to improve adhesion to the Si substrate. Since the compliance of the grip head (C_{grip}) and the compliance of the base (C_{base}) cannot be assumed to be infinitely small compared to the compliance of the lattice sample, the vertical displacement of the lattice (u_{lat}) needs to be corrected as follows:

$$u_{total} = u_{lat} + u_{grip} + u_{base}$$

$$u_{total} = \left(C_{lat} + C_{grip} + C_{base}\right) * Load,$$

where u_{total}, u_{grip}, and u_{base} are the displacements of the nanoindenter, the grip head, and the base, respectively, and C_{lat} is the lattice compliance. Here, the nanoindenter (labeled as machine) and the Si substrate are assumed to be rigid with respect to the IP-Dip components. Using the stiffness-compliance relationship $K_{eff} = \frac{1}{C_{grip} + C_{base}}$,

$$u_{lat} = u_{total} - \left(\frac{1}{K_{eff}} * Load\right). \qquad \textbf{[Equation S1]}$$

As shown in Figure S13, the stiffness of the combined grip head and base was measured by loading the grip head and base alone without any lattice (or pillar) in between. There are three different grip categories: grips for tension-to-compression lattice experiments, tension-only lattice experiments, and tension-only IP-Dip pillar experiments. Each grip category for lattices consists of one grip design for octahedron lattices and one for diamond lattices; there are a total of five grip designs in this work. Each unit cell configuration merits its own grip design because the grip is susceptible to printing failure in regions where it is not connected to the beams of the lattice underneath it (Figure S14). We have measured the stiffness of each grip design and obtained K_{eff} values ranging from 9.7 to 16.8 kN m^{-1} depending on which unit cell and loading configuration the lattice grip head was designed for. For pillar samples, the average K_{eff} value was 3.7 kN m^{-1}.

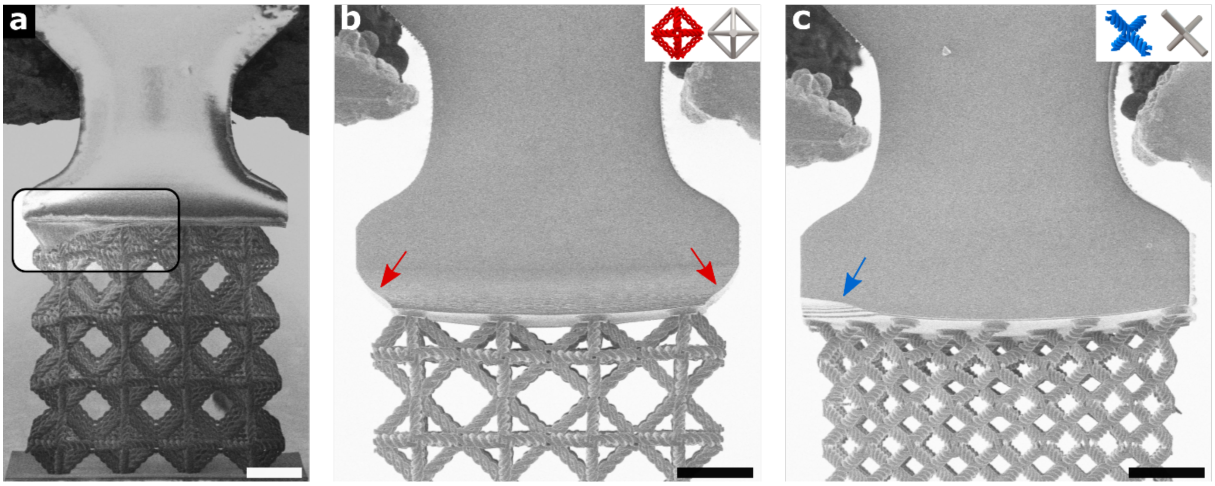

Figure S14. Grip design for each unit cell architecture. a) Printing failure shown in a grip head designed for tension-only experiments. For each unit cell architecture, the region of the grip where the grip meets the sample was customized to prevent printing failure. Red and blue arrows point to major differences between grips intended for b) octahedron and c) diamond unit cell architectures, respectively. Scale bars are 25 µm.

Numerical Study Methods

We performed linear perturbation simulations of a 3-fiber cantilever woven strut with varying geometric parameters. Each fiber was constrained to undergo one full rotation (i.e., the pitch was the same as the strut length L), and the effective beam radius was solved for to attain a given solid volume (determined by a choice of fiber radius and a comparison monolithic beam). The fibers were meshed using 10-node quadratic tetrahedral elements (C3D10), with a minimum of 4 elements across the fiber diameter, corresponding to 3,000-24,000 elements per fiber depending on geometric features. The DOFs of the nodes on one side of the fibers were fixed, while the DOFs on the opposite side were coupled to a single dummy node for all three fibers. A displacement was then imposed on the dummy node, as shown in Figure 3e,f, for the stretching and bending cases. The normalized effective axial and bending stiffnesses were computed by dividing the reaction force at the dummy node by the displacement amplitude, and then normalizing by EL, where E is the Young's modulus. To directly compare to their monolithic counterparts, these normalized effective axial and bending stiffnesses were divided by the corresponding normalized stiffnesses of the monolithic beams, and are presented as surface plots in Figure 3e,f.

Effects of Prolonged UV Light Exposure in Samples for Tension Experiments

While one out of every three samples tested in tension for each architecture and unit cell configuration was tested within three days after fabrication, the other two samples were tested within four to five months after fabrication. The delay in testing may have led to increased cross-linking of IP-Dip due to UV light exposure over an extended amount of time.[3] Interestingly, the only type of architecture that produced a considerable change in response due to delay in testing was monolithic octahedron, where $\sigma_{t,f}$ increased but $\varepsilon_{t,f}$ did not noticeably change (see Figure 2a,b). Note that the monolithic octahedron architecture is the only lattice type in this work that does not have a non-rigid hierarchy whether in the unit cell-level or beam-level, and it is safe to assume that the r/L value (which corresponds to $\bar{\rho} \approx 5\ \%$) is low enough such that the architecture is still stretching-dominated.[2,4]

References

[1] J. Bauer, A. Guell Izard, Y. Zhang, T. Baldacchini, L. Valdevit, *Adv. Mater. Technol.* **2019**, *4*, 1.
[2] C. M. Portela, J. R. Greer, D. M. Kochmann, *Extrem. Mech. Lett.* **2018**, *22*, 138.
[3] J. S. Oakdale, J. Ye, W. L. Smith, J. Biener, *Opt. Express* **2016**, *24*, 27077.
[4] L. R. Meza, G. P. Phlipot, C. M. Portela, A. Maggi, L. C. Montemayor, A. Comella, D. M. Kochmann, J. R. Greer, *Acta Mater.* **2017**, *140*, 424.
